# Tip60/KAT5 Histone Acetyltransferase Is Required for Maintenance and Neurogenesis of Embryonic Neural Stem Cells

**DOI:** 10.3390/ijms24032113

**Published:** 2023-01-20

**Authors:** Kaoru Tominaga, Eiji Sakashita, Katsumi Kasashima, Kenji Kuroiwa, Yasumitsu Nagao, Naoki Iwamori, Hitoshi Endo

**Affiliations:** 1 Division of Structural Biochemistry, Department of Biochemistry, Jichi Medical University, Tochigi 321-0498, Japan; 2 Division of Functional Biochemistry, Department of Biochemistry, Jichi Medical University, Tochigi 321-0498, Japan; 3 Center for Experimental Medicine, Jichi Medical University, Tochigi 321-0498, Japan; 4 Department of Agriculture, Kyushu University, Fukuoka 819-0395, Japan

**Keywords:** neural stem cell, Tip60/KAT5, neurogenesis, gliogenesis, acetyltransferase, epigenetics

## Abstract

Epigenetic regulation via epigenetic factors in collaboration with tissue-specific transcription factors is curtail for establishing functional organ systems during development. Brain development is tightly regulated by epigenetic factors, which are coordinately activated or inactivated during processes, and their dysregulation is linked to brain abnormalities and intellectual disability. However, the precise mechanism of epigenetic regulation in brain development and neurogenesis remains largely unknown. Here, we show that Tip60/KAT5 deletion in neural stem/progenitor cells (NSCs) in mice results in multiple abnormalities of brain development. Tip60-deficient embryonic brain led to microcephaly, and proliferating cells in the developing brain were reduced by Tip60 deficiency. In addition, neural differentiation and neuronal migration were severely affected in Tip60-deficient brains. Following neurogenesis in developing brains, gliogenesis started from the earlier stage of development in Tip60-deficient brains, indicating that Tip60 is involved in switching from neurogenesis to gliogenesis during brain development. It was also confirmed in vitro that poor neurosphere formation, proliferation defects, neural differentiation defects, and accelerated astrocytic differentiation in mutant NSCs are derived from Tip60-deficient embryonic brains. This study uncovers the critical role of Tip60 in brain development and NSC maintenance and function in vivo and in vitro.

## 1. Introduction

Epigenetic regulations through DNA methylation, histone post-translational modifications, and non-coding RNAs have been widely investigated [1,2,3] and recognized as critical factors in neurogenesis and brain development [4,5,6,7,8,9]. The dysregulation of epigenetic mechanisms has been linked to neurological disorders such as autism, epilepsy, intellectual disability, and neurodegenerative diseases such as Alzheimer’s disease (AD) [10,11,12,13,14]. Histone acetylation is a major epigenetic modification, and it is involved in many cellular processes [15,16,17,18,19]. Histone acetylation destabilizes DNA–histone interactions and forms an open chromatin state. The kinetics of proper histone acetylation are maintained by the action of histone acetyltransferases (HATs) and deacetylases (HDACs), and open or closed chromatin states are properly coordinated to regulate DNA-related processes such as gene expression and DNA repair.

Tip60/KAT5 is a member of the MYST (Moz, Ybf2/Sas3, Sas2, and Tip60) family HAT, and its major acetylation sites include lysine (K)5, 8, and 12 on histone H4 and K5 on histone H2A and histone variant H2AX [20,21,22]. At present, Tip60 participates in many cellular processes such as transcriptional regulation, cell cycle control, apoptosis, autophagy, and DNA repair [23,24,25,26,27]. Based on previous reports, Tip60 expression is high in the hippocampal CA1 region in adult mice [28]. Tamoxifen-induced conditional *Tip60* deficiency in postmitotic excitatory neurons of the adult forebrain reduced H4K12 acetylation and the dysregulation of gene expression in the hippocampal CA1 region [29]. Inflammation and neurodegeneration in the CA1 region gradually progressed through *Tip60* deficiency, and *Tip60*-deficient mice showed modest memory impairment at a later time point [29]. Adeno-associated virus-mediated *Tip60* knockdown (KD) in the hippocampal CA1 region in adult mice showed impairment of stimulation-dependent synaptic plasticity and long-term memory formation [30]. This impairment was correlated with the suppression of Fgf1b expression following the decrease in H4K12 acetylation on the Fgf1b promoter. This finding indicated that Tip60-mediated histone acetylation is involved in the upregulation of specific target genes for training-induced enduring memory formation in the hippocampus. Recently, three individuals with heterozygous de novo missense variants in Tip60/KAT5 were reported [31]. These patients showed developmental delay, cerebellar malformation, seizures, intellectual disability, sleep disturbance, and epilepsy. The mutations were found in the chromodomain (p.Arg53His) and in the acetyl-CoA-binding region (p.Cys369Ser and p.Ser413Ala). All amino acids are evolutionally well conserved, and all mutations reduced histone acetylation activities when native nucleosomes were used as a substrate. Therefore, Tip60 activity might be essential for neurogenesis and neuronal functions.

In a *Drosophila* AD model, the dysregulation of balance between Tip60 and HDAC2 reduced neuroplasticity and disease progression [32,33,34,35]. The acetylation homeostasis in AD brains is impaired because of the loss of Tip60 HAT activity and the gain of HDAC2 activity in this model, which disrupts the expression of neuroplasticity genes and may accelerate the progression of AD pathogenesis. In addition, the reinforcement of Tip60 activity in the brain restores the expression of neuroplasticity genes and brain health in this model. Evidence that Tip60 HAT is an essential factor for neuronal functions is accumulating, but whether Tip60 is involved in the maintenance of neural stem/progenitor cells (NSCs) and neurogenesis, particularly in the fetal brain, remains unclear.

In order to obtain insights into the role of Tip60 in NSC maintenance and neurogenesis in the mammalian brain, we have generated a NSC-specific *Tip60* knockout mouse model in which Cre recombinase is expressed under the control of the Nestin promoter active in NSCs. Our findings indicate that Tip60 plays a critical role in NSC maintenance, neurogenesis, and switching from neurogenesis to gliogenesis during mouse brain development.

## 2. Results

### 2.1. Tip60 Deletion in Neural Stem Cells Causes Perinatal Lethality

**Figure 1:**
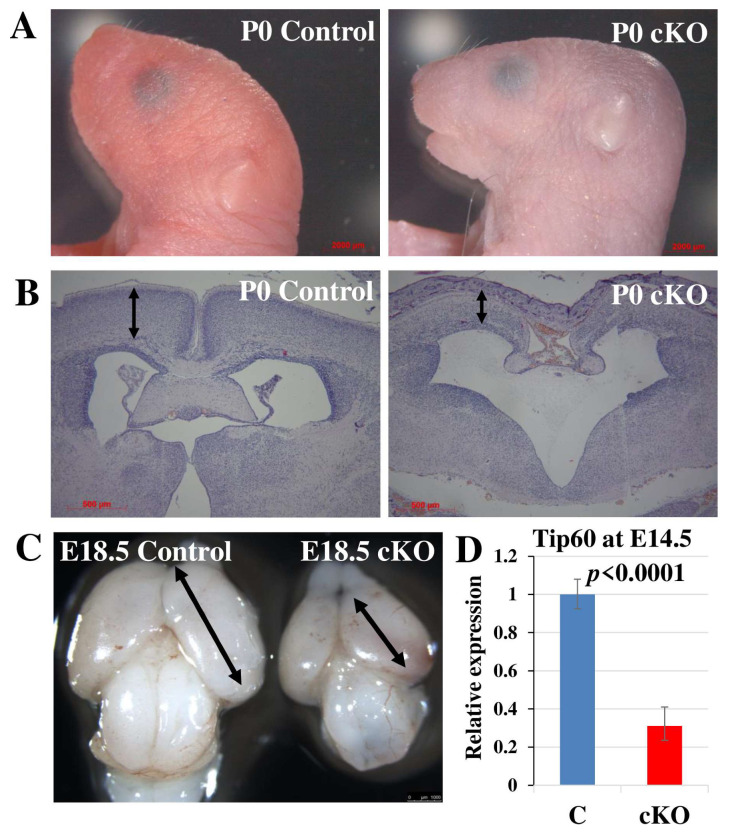
*Tip60* inactivation in NSCs results in microcephaly and perinatal lethality. (**A**) Representative images of head parts of the mice at P0. *Tip60* cKO pup shows the flat-head phenotype. Scale bars, 2000 µm. (**B**) H and E staining of coronal brain sections at P0. The arrow indicates the cerebral cortex. Scale bars, 500 µm. (**C**) Representative brain images for control and *Tip60* cKO at E18.5. The cerebral cortex of *Tip60* cKO is smaller than that of control. Scale bar, 1000 µm. (**D**) Relative expression of Tip60 mRNA in the cerebral cortex at E14.5 measured by qRT-PCR. The expression levels of Tip60 mRNA were normalized with the expression levels of β-actin. The graph shows means ± SD. *n* = 3 brains per group. At least two independent experiments were performed.

In investigating Tip60 function in neurogenesis in vivo, we produced NSC-specific *Tip60*-deficient mice by using the *Nes-Cre^+^* strain, in which Cre recombinase is expressed in neural stem cells starting at E11.5 (Figure S1) [36]. These *Tip60^F/+^;Nes-Cre^+^* males were crossed with *Tip60^F/F^* females to produce *Tip60^F/F^;Nes-Cre^+^* (*Tip60* cKO) mice. Although the *Tip60* cKO mice were born at the expected Mendelian ratio (Table 1), we did not obtain any viable *Tip60* cKO animals after birth, probably because of respiratory distress. At birth (P0), *Tip60* cKO mice showed a flat-head phenotype compared with wild-type mice (Figure 1A). Histopathological analysis of the P0 brain showed that the cortex of the *Tip60* cKO was thinner, and its brain size was smaller than that of the wild-type (Figure 1B). The deletion of Tip60 in NSCs caused severe hypoplasia of the brain and microcephaly, and this phenotype was evident even at the embryonic stage E18.5 (Figure 1C). We also examined the expression of Tip60 in the brain of *Tip60* cKO embryos. We selected E14.5 embryos for the detection of Tip60 expression because their Cre recombinase is already functional at this embryonic stage. As expected, the expression level of Tip60 in the cortex of *Tip60* cKO was significantly reduced even at E14.5 compared with that of the control (Figure 1D). The level of histone acetylation at H4Lys8 and H4Lys12 (H4K8Ac and H4K12Ac), which are reported as the target sites of Tip60, was reduced in the cerebral cortex of *Tip60* cKO embryonic brains at E16.5 (Figure S2).

Considering that Tip60 is involved in DNA damage response and repair, we investigated DNA damage response and apoptosis in the *Tip60* cKO brain to elucidate the perinatal lethality and cause of microcephaly. Thus, we performed immunohistochemistry (IHC) of the brain at E18.5 using antibodies against cleaved caspase 3 for the detection of apoptosis and γH2AX for the detection of DNA damage (Figure S3A). Although positive cells for cleaved caspase 3 and γH2AX could not be detected in the control brain, γH2AX-positive cells in the Tip60 cKO brain, particularly at the subventricular zone (SVZ), were accumulated. In addition, only a few apoptotic cells, which were positive for cleaved caspase 3, could be detected in the *Tip60* cKO brain, but the number of apoptotic cells was fewer than what we expected. The possibility of accelerated apoptosis may not cause microcephaly of the *Tip60* cKO brain at least until E18.5. The accumulation of γH2AX-positive cells was also evident in the *Tip60* cKO brain at E16.5 (Figure S3B). Double-positive cells for γH2AX and 53BP1, another marker for DNA damage, could not be detected at SVZ in the *Tip60* cKO brain at E16.5. Next, we examined the expression of p21, which is a well-known downstream target of p53 in response to DNA damage. Accumulated p21 expression could not be detected in the Tip60 cKO brain at E18.5, although γH2AX-positive cells were easily detected (Figure S3C). The p21-dependent cellular senescence program may not be activated in *Tip60* cKO brain. We did not perform any further investigations relating to DNA damage in this study.

### 2.2. Tip60 Deletion in NSCs Causes Abnormalities in Neurogenesis and Neuronal Migration

**Figure 2:**
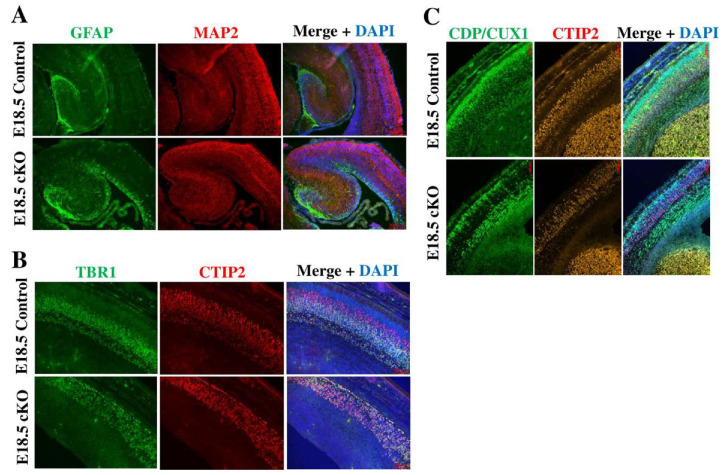
Tip60 deficiency shows decreased neurogenesis, increased gliogenesis, and abnormal neuronal migration in developing brains. (**A**) Representative images of brains at E18.5 stained for GFAP (astrocytes) and MAP2 (neurons) by immunofluorescence. DAPI was used for nuclear staining. Scale bars, 200 µm. (**B**) Representative images of brains at E18.5 stained for layer-specific marker proteins, TBR1 (Layers I and VI), and CTIP2 (Layer V). DAPI was used for nuclear staining. Scale bars, 100 µm. (**C**) Representative images of brains at E18.5 stained for layer-specific marker proteins, CUX1 (Layer II/III), and CTIP2 (Layer V). DAPI was used for nuclear staining. Scale bars, 100 µm. Staining results were confirmed by at least two independent experiments.

In addition, we investigated the effect of Tip60 deficiency on neurogenesis using immunohistochemical analyses. We used E18.5 embryos for these analyses. All *Tip60* cKOs at E18.5 were alive, although the body size of *Tip60* cKO was slightly smaller than the control. First, we used antibodies against MAP2 and GFAP to detect mature neurons and astroglia, respectively. The MAP2-positive neuronal population of the *Tip60* cKO brain was smaller than that of the control brain, and MAP2-positive cells in the *Tip60* cKO brain were disorganized compared with those in the control brain (Figure 2A,B). GFAP-positive cells in the control brain were detected only in the hippocampus, whereas GFAP-positive cells in the *Tip60* cKO brain were detected in the ventricular zone and hippocampus at E18.5. Next, we used antibodies against layer-specific neuronal markers, such as TBR1 (layer VI), CTIP2 (layer V), and CDP/CUX1 (layers II/III and IV), to evaluate neurogenesis and neuronal migration [37,38]. As shown in Figure 2C, TBR1-positive and CTIP2-positive neurons properly migrated in the control brain, whereas TBR1-positive neurons and CTIP2-positive neurons were located at the same place in the *Tip60* cKO brain. A part of CDP/CUX1-positive neurons in the *Tip60* cKO brain properly migrated into the cortex, but the majority of CDP/CUX1-positive neurons did not migrate and stayed under layer VI. On the contrary, CDP/CUX1-positive neurons in the control brain migrated to the expected position. These results indicated that Tip60 is involved in neuronal maturation during neurogenesis and the impairment of Tip60 function in neurogenesis, thereby causing a disorganized brain structure.

### 2.3. Loss of Tip60 in NSCs Shows Cell Proliferation Defect and Accumulation of M Phase Cells in the Brain

**Figure 3:**
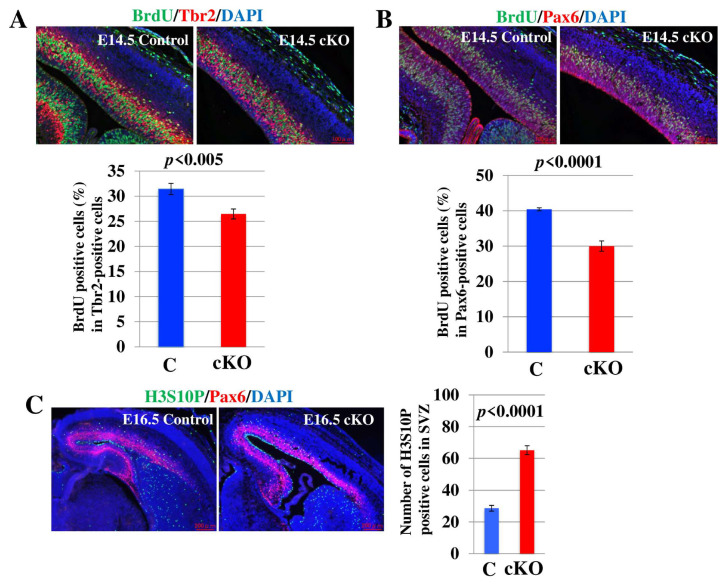
*Tip60-*deficient brains show proliferation defects and cell cycle dysregulation. (**A**) Representative images of brains at E14.5 stained for BrdU and Tbr2 (markers for BPs) by immunofluorescence. DAPI was used for nuclear staining. Scale bars, 100 µm. The ratio of BrdU-positive and -negative cells in Tbr2-positive cells is shown. At least 10 different sections were counted. The graph shows means ± SD. Two independent experiments were performed. (**B**) Representative images of brains at E14.5 stained for BrdU and Pax6 (marker for APs) by immunofluorescence. DAPI was used for nuclear staining. Scale bars, 100 µm. The ratio of BrdU-positive and -negative cells in Pax6-positive cells is shown. At least 10 different sections were counted. The graph shows means ± SD. Two independent experiments were performed. (**C**) Representative images of brains at E16.5 stained for H3S10P (marker for mitosis) and Pax6 by immunofluorescence. DAPI was used for nuclear staining. Scale bars, 200 µm. The number of H3S10P-positive cells in SVZ is counted. At least 10 different sections were counted. The graph shows means ± SD. At least two independent experiments were performed.

In order to verify whether the loss of Tip60 in NSCs causes microcephaly, we performed a BrdU incorporation assay in the brain at E14.5. BrdU-incorporated cells were detected by IHC using an anti-BrdU antibody, concomitant with detection using an antibody against Tbr2, a basal progenitor (BP) marker (Figure 3A) or Pax6, an apical progenitor (AP) marker (Figure 3B) [39]. The ratio of BrdU-positive cells in Tbr2-positive cells in the *Tip60* cKO cortex was significantly reduced compared to that in the control cortex (Figure 3A). Similar to Tbr2-positive cells, the ratio of BrdU-positive cells in Pax6-positive cells in *Tip60* cKO cortex was also significantly reduced compared with that in the control cortex (Figure 3B). Based on previous reports, Tip60 is involved in the proper progression of mitosis in cancer cells. We performed IHC of E16.5 brains using an antibody against phosphorylated histone H3 at Serine 10 (H3S10P), which is a marker for mitosis. The expression of H3S10P-positive cells in SVZ in the *Tip60* cKO brain was evidently decreased compared to that in control brain. The accumulation of H3S10P-positive cells in SVZ in the *Tip60* cKO brain might reflect the increase in Tbr2-positive cells. These results indicated that the loss of Tip60 in NSCs affected cell growth in the brain during neurogenesis.

### 2.4. Loss of Tip60 in NSCs Affects Cell Growth and Differentiation In Vitro

**Figure 4:**
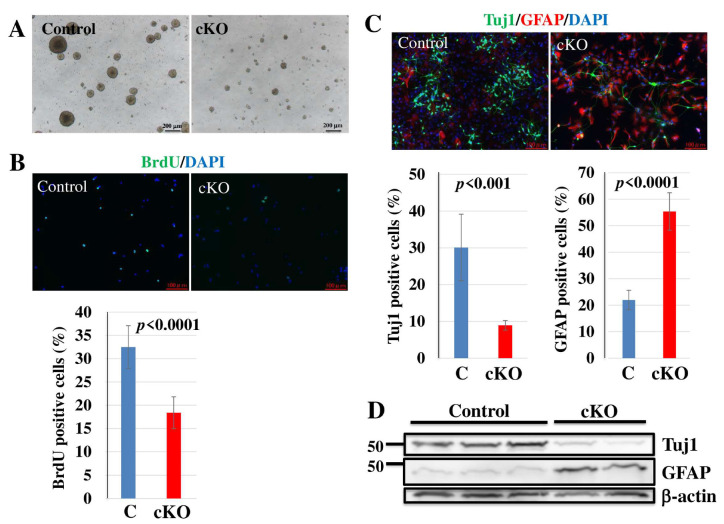
Critical roles of Tip60 in neurosphere formation, proliferation, and neural differentiation in vitro. (**A**) A neurosphere formation defect in NSCs derived from *Tip60*-deficient cerebral cortex but not from control at E14.5. These representative pictures were taken after a 6-day culture. Scale bars, 200 µm. (**B**) Proliferation assay. Cultured NSCs were incubated with 10 µM BrdU for 2 h, and the incorporated BrdU was detected with an anti-BrdU antibody. DAPI was used for nuclear staining. Scale bars, 100 µm. BrdU-positive cells were counted. The graph shows means ± SD. At least two independent experiments were performed. (**C**) Differentiation assay. NSCs were cultured in a differentiation medium for 7 days. Neurons and astrocytes were detected by immunostaining with anti-Tuj1 and anti-GFAP antibodies, respectively. DAPI was used for nuclear staining. Scale bars, 100 µm. Tuj1 and GFAP-positive cells were counted. The graph shows means ± SD. At least three independent experiments were performed. (**D**) Western blot analysis of Tuj1 and GFAP for in vitro-differentiated NSCs. NSCs were cultured in a differentiation medium for 7 days, and total cell lysates were prepared. The expression of Tuj1 and GFAP was detected by specific antibodies. Beta-actin was used as an internal control. Two independent experiments were performed.

In investigating the role of Tip60 in cell growth and differentiation of NSCs in vitro, we performed in vitro neurosphere cultures and used them for further in vitro studies. As shown in Figure 4A, the sphere size in *Tip60* cKO culture was smaller than that in control culture. Similar to sphere size distribution, BrdU incorporation in *Tip60* cKO NSCs was significantly reduced compared with that in control. This result indicated that Tip60 is important for cell growth in NSCs, which was confirmed in vivo and in vitro.

Moreover, we investigated the role of Tip60 in the differentiation ability of NSCs in vitro. NSCs were cultured in a differentiation medium without mitogens for 7 days, and neurons and astrocytes were detected by ICC using antibodies against Tuj1 and GFAP, respectively. Similar to in vivo studies (Figure 2A), where the expression of Tuj1-positive neurons in *Tip60* cKO significantly decreased compared to those in control, GFAP-positive astrocytes in *Tip60* cKO significantly increased (Figure 4C). This result was also confirmed by Western blot (Figure 4D). Tuj1 expression in *Tip60* cKO cultures was significantly lower than that in control cultures. On the contrary, GFAP expression in *Tip60* cKO cultures was significantly higher than that in control cultures.

### 2.5. Microglia Are Activated in Tip60 cKO Brain

**Figure 5:**
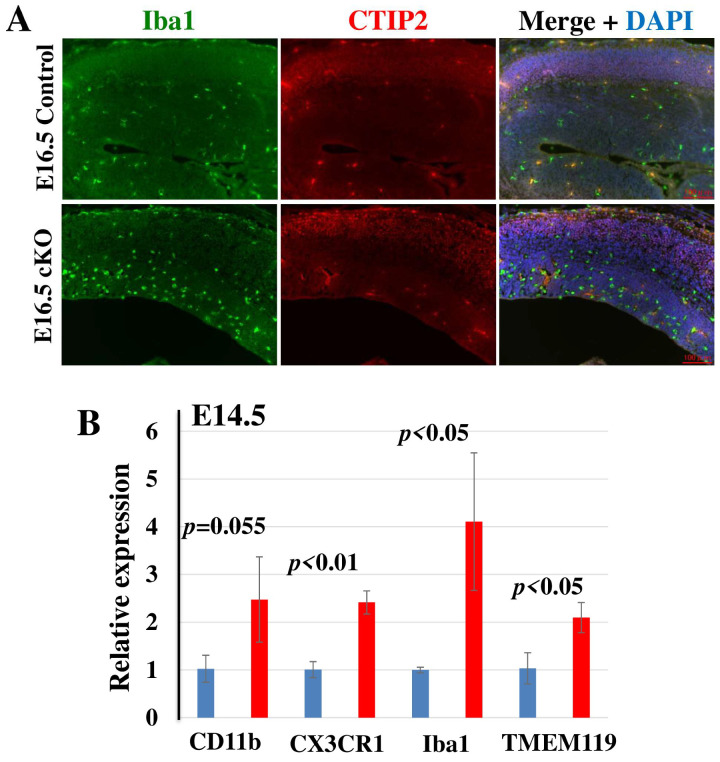
Microglial activation in *Tip60*-deficient brains. (**A**) Representative images of immunostaining for Iba1 (microglia) and CTIP2 (Layer V) with DAPI nuclear staining of brains at E16.5. Scale bars, 100 µm. Iba1-positive activated microglia are more detected in *Tip60* cKO brains compared to control brains. (**B**) The expression of microglia marker genes. The expression levels of some microglia marker genes in E14.5 brains were examined by qRT-PCR. These expression levels were significantly upregulated in *Tip60* cKO brains compared with those in control brains. The graph shows means ± SD. *n* = 3 mice per group.

Microglia are activated in response to physiological and pathophysiological insults, and they are involved in the maintenance of proper brain function. In elucidating the microglial status in the *Tip60* cKO brain, we conducted IHC using an antibody against Iba1, a microglial marker [40], concomitant with an anti-CTIP2 antibody, a neuronal marker for Layer V of the cortex. As shown in Figure 5A, the number of Iba1-positive microglia in the *Tip60* cKO brain at E16.5 evidently increased compared to that in control brain. As microglia are activated by extracellular stimulation, they are transformed from a ramified (resting form) to an ameboid (active form) morphology. Many microglia showed ameboid morphology in the *Tip60* cKO brain at E16.5, whereas almost all microglia in the control brain had ramified morphology. The number of microglia increased, and they were activated in the *Tip60* cKO brain at E16.5. Although microglia at this stage can be detected only in VZ/SVZ [41,42], Iba1-positive microglia in the *Tip60* cKO brain were also recognized in the cortex. The expression level of microglial marker genes in the *Tip60* cKO brain significantly increased compared to that in the control brain even at E14.5 (Figure 5B). These results indicated that the effects of the loss of Tip60 in the brain had appeared as early as E14.5.

### 2.6. Tip60 Regulates Neurogenesis in Developing Brain

**Figure 6:**
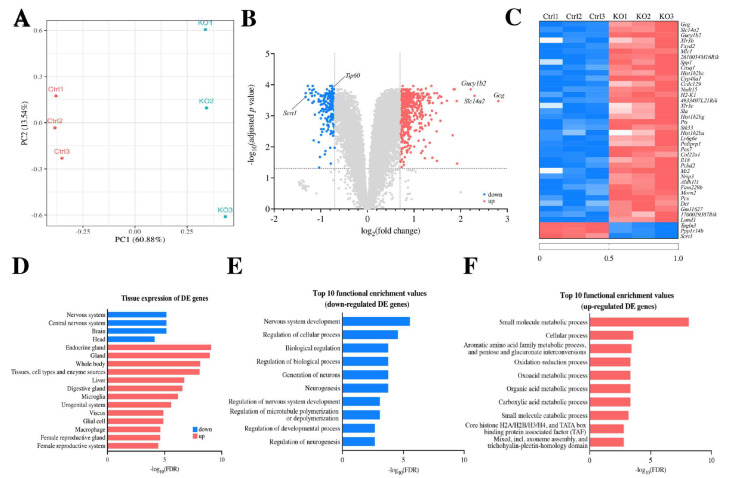
Tip60 is required for neurogenesis. (**A**) PCA plot of six RNA-seq samples (three *Tip60* cKO and three control brains at E14.5) based on the expression level (RPKM) of 20,079 protein-coding genes. The proportion of variance explained is indicated in parentheses. (**B**) Volcano plot of statistically significant and differentially expressed genes (DE genes) at adjusted *p* value < 0.05 and fold changes ≥1.5. (**C**) Heatmap of the top 40 DE genes by fold change in *Tip60* cKO vs. control groups. (**D**) Tissue expression enrichment for 208 downregulated and 547 upregulated DE genes. (**E**) Top 10 functional enrichment values for DE genes downregulated in *Tip60* cKO. (**F**) Top 10 functional enrichment values for DE genes upregulated in *Tip60* cKO.

In exploring genes affected by Tip60 deficiency during neurogenesis, we conducted RNA-seq analysis using mRNA isolated from brain cortexes at E14.5 and compared the gene expression pattern in *Tip60* cKO embryonic cerebral cortexes with that in control cerebral cortexes. Considering that abnormalities in the brain caused by Tip60 deficiency became evident right after E14.5, we selected brains at this stage for RNA-seq analyses. Principal component analysis (PCA) based on the expression level of over 20,000 protein-coding genes demonstrated that the gene expression signature between two groups was clearly separated along the PC1 axis (Figure 6A). The distribution of differentially expressed (DE) genes between *Tip60* cKO and control groups is depicted by a volcano plot (Figure 6B). The expression patterns of the top 40 DE genes are illustrated in a heatmap of three replicates of each group (Figure 6C). Tissue expression enrichment analysis was performed using 208 downregulated and 547 upregulated gene sets (Figure 6D). The genes categorized in the nervous system and brain were downregulated in *Tip60* cKO, whereas the genes categorized in microglia and glial cells were upregulated in *Tip60* cKO. This result completely overlapped with the result related to microglial activation in the *Tip60* cKO brain (Figure 5). The top 10 functional categories for DE genes on enrichment analysis, including the biological process gene ontology (GO) term and STRING cluster, are indicated in Figure 6E (downregulated genes) and 6F (upregulated genes). The majority of the downregulated genes in *Tip60* cKO cortexes were listed in multiple pathways important for neurogenesis and nervous system development. Scratch 1 (Scrt1), which was located at the top rank in the downregulated gene list in *Tip60* cKO cerebral cortex, regulates neuronal migration via an epithelial–mesenchymal transition-like mechanism [43,44], and abnormal migration of neuronal cells in *Tip60*-deficient cerebral cortex during development might be related to the downregulation of Srct1 expression. These data also suggest that Tip60 is a critical regulator for neural stem cell maintenance and neurogenesis.

### 2.7. Glucagon-like Peptide-1 (GLP-1) Positive Cell Population Is Increased in Tip60 cKO Brains

**Figure 7:**
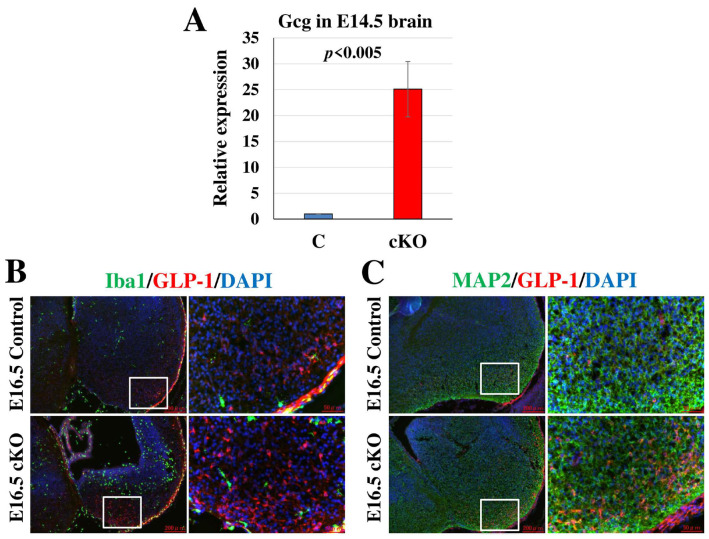
Tip60 deficiency in the brain increases the GLP-1-expressed neural population. (**A**) The relative expression level of the *Gcg* gene. The relative expression level of the *Gcg* gene in *Tip60* cKO brains at E14.5 was determined using qRT-PCR and compared with control brains. The expression level of β-actin was used as an internal control. (**B**) Representative images of immunostaining for Iba1 (microglia) and GLP-1 with DAPI nuclear staining of brains at E16.5. Enlarged images of the boxed areas are shown at the right. Scale bars, 200 µm, 50 µm in enlarged view. (**C**) Representative images of immunostaining for MAP2 (Neuron) and GLP-1 with DAPI nuclear staining of the brains at E16.5. Enlarged images of the boxed areas are shown at the right. Scale bars, 200 µm, 50 µm in enlarged view.

The most strikingly upregulated gene in *Tip60*-deficient brains by RNA-seq analysis was the preproglucagon (Gcg) gene, which was confirmed by qRT-PCR (Figure 7A). Proglucagon is differentially processed by prohormone convertases, which produce several different peptides such as glucagon and GLP-1 in a tissue-specific manner [45,46]. Based on previous reports, GLP-1 is expressed in amoeboid-type activated microglia, and activated microglia [47,48] were accumulated in *Tip60*-deficient embryonic brains (Figure 5). Thus, we examined whether GLP-1 is expressed in activated microglia in *Tip60*-deficient embryonic brains at E16.5 using IHC. As shown in Figure 7B, although we could detect an increased number of GLP-1-positive cells in the *Tip60*-deficient embryonic brain compared with the control brain, GLP-1-positive staining did not overlap with Iba1-positive microglia. Next, we performed co-immunostaining for GLP-1 and MAP2, which are neuronal markers. As shown in Figure 7C, GLP-1 staining overlapped with MAP2-positive cells. This finding indicated that GLP-1 was expressed in some types of neurons. Considering that the number of GLP-1-positive cells but not the levels of GLP-1 expression in each cell increased in *Tip60*-deficient brains, Tip60 might be involved in the specification of some types of neuronal linages.

## 3. Discussion

**Figure 8:**
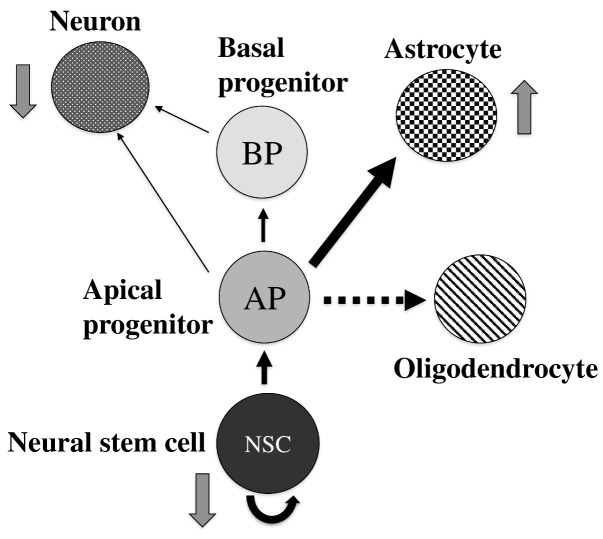
Schematic illustration of the role of Tip60 in NSC maintenance and neurogenesis.

In this study, we explored the role of Tip60 HAT in the maintenance of neural stem cells and embryonal neurogenesis using a NSC-specific *Tip60* knockout mouse model. NSC-specific *Tip60* cKO mice were born at a Mendelian ratio, but they died soon after delivery. *Tip60* cKO mice showed microcephaly, and NSCs derived from *Tip60* cKO brains showed a proliferation defect in in vivo and in vitro accumulation of M phase cells in vivo, indicating that the dysregulation of cell growth in *Tip60*-deficient NSCs contributes to the occurrence of microcephaly in the *Tip60* cKO brain. Neuronal differentiation and migration were severely affected in the brains of *Tip60* cKO mice, whereas GFAP-positive glial cells increased in the embryonal brain during in vivo and in vitro differentiation using neurosphere cultures derived from *Tip60* cKO mice. Gene expression analyses also showed that the expression of genes related to multiple neuronal pathways was downregulated in *Tip60*-deficient embryonal brains. The role of Tip60 in NSCs and neurogenesis, which is speculated from this study, is summarized in Figure 8.

M-phase cells in the cell cycle, identified by the detection of phosphorylated histone H3 at Ser10, were accumulated in SVZ and most likely in VZ in *Tip60*-deficient embryonic brains. Recently, Rust et al. reported that the Tip60 complex controls *Drosophila* neuroblast maintenance and polarity via interaction with Myc [49]. The depletion of ATPase Domino (dom), one of the subunits of the Tip60 complex, in neuroblasts showed abnormalities in spindle morphology and spindle asymmetry, which might cause aberrant division of dom-depleted neuroblasts. Messina et al. have reported that the subunits of the p400/Tip60 complex relocate from the interphase chromatin to the mitotic apparatus, and this dynamic relocation is necessary for the execution of proper cell division [50]. This regulation is evolutionarily conserved from fly to human. Tip60 is also known to be involved in protein modifications for several critical targets, such as acetylation and crotonylation, to execute accurate mitosis in several cancer cells [51,52,53,54,55,56]. In this study, mitotic cells were accumulated in the SVZ and most likely in the VZ of *Tip60*-deficient embryonic brains. This finding suggests that the regulation of mitosis by the Tip60 complex is crucial for brain development and neurogenesis.

Tip60 is also important for S-phase entry of the cell cycle to control the expression of E2F target genes, such as PCNA and cdc25A [57,58]. BrdU incorporation into *Tip60*-deficient embryonic brains and NSCs cultured in vitro was significantly reduced. This in vivo observation highly reflects previous in vitro observations. Interestingly, apoptosis detected by activated caspase 3 and senescence detected by p21 expression were not clearly upregulated in *Tip60*-deficient brains. Recently, Wichmann et al. have reported similar observations using *Tip60*-deficient human cancer cells and mouse embryonic fibroblasts [59]. They showed that the loss of Tip60 causes complete cell growth arrest without affecting cell survival, and the cell growth arrest caused by the loss of Tip60 was independent of p53, INK4A, and ARF. This finding suggests that microcephaly in *Tip60*-deficient mouse embryos is primarily due to inefficient cell growth, and cell growth arrest is due to the loss of Tip60.

NSCs promote the generation of neurons and glial cells (astrocytes and oligodendrocytes). During development, neurons are generated at early embryonic stages around E10.5, whereas astrocytes start to be generated at E18.5 or later stages in rodents [60,61,62]. Switching from neurogenesis to astrogenesis in NSCs is tightly regulated by intrinsic and extrinsic factors to ensure a proper balance between neuronal and glial cells [63,64]. Astrogenesis and astrocyte differentiation were accelerated in *Tip60*-deficient embryonic brains and in vitro differentiation of NSC culture. Similarly, the deletion of the histone methyltransferase Ezh2 of PRC2, which is crucial for the removal of the repression mark of trimethylated histone H3 at lysine 27 (H3K27me3), results in the appearance of GFAP-positive astrocytes in the cerebral cortex at E16 when astrocytes are not normally found [65]. PRC2 tightly controls the developmental timing of the transition from neurogenesis to astrogenesis, and Tip60 might also be a crucial factor in this regulation. The JAK-STAT3 signaling pathway is important for astrogenesis in late embryogenesis [66,67]. STAT3, phosphorylated by activated JAKs, is translocated into the nucleus, and it activates astrocytic gene expression, including GFAP, thereby promoting astrogenesis. Tip60 interacts with STAT3 and serves as a co-repressor for STAT3 in cancer cells via recruitment of HDAC7 to STAT3 target genes [68]. Tip60 might interact with STAT3 and repress the astrocytic gene expression controlled by STAT3 in NSCs. STAT3 might effectively interact with co-activator p300/CBP [66] and could activate the expression of astrocytic target genes such as GFAP in a Tip60-deficient condition.

The *preproglucagon* gene (*Gcg*) encodes some different bioactive polypeptides, such as glucagon and GLP-1, and the production of final peptides is cell type dependent [45,46]. In the present study, we identified Gcg as the most significantly upregulated gene in *Tip60*-deficient embryonic brains and confirmed that the number of GLP-1 positive neurons increased in the piriform cortex, in which GLP-1 expression was suggested [46,69] in *Tip60*-deficient embryonic brains through an immunohistological analysis. The number of GLP-1-positive neurons during brain development might be controlled by Tip60 activities, and Tip60 might also be involved in the specification of some specific neuronal linages during neurogenesis. Considering that GLP-1 signaling in the brain is linked to physiological activities in animals, such as food consumption [70,71,72], further investigation is recommended. In general, histone acetylation is linked to the transcriptional activation of target genes. However, Tip60 primarily serves as a transcriptional repressor in ESCs [73,74]. Recently, the Tip60/p400 complex, in collaboration with Rap1, repressed the expression of a subset of two-cell-stage genes such as Zscan4 and the endogenous retrovirus MERVL in ESCs [75]. Chen et al. have shown that HDAC6 interacts with the Tip60/p400 complex and represses a subset of differentiation-related genes in ESCs [76]. Whether Gcg is a direct target of Tip60 in developing brain has not been investigated to date.

The role of several acetyltransferases has been investigated during brain development and linked to neurodevelopmental disorders. CBP/p300 family members are essential for proper brain development, and genetic mutations in these proteins are related to Rubinstein–Taybi syndrome with learning disability [77,78]. It is also reported that haploinsufficiency in KAT8/Mof leads to a cerebral development anomaly and an intellectual disability. Similar to brain-specific *Tip60*-deficient mice, cerebrum-specific *KAT8*-deficient mice using the *Emx1-Cre* line had severe cerebral hypoplasia with impaired cell proliferation and aberrant neurogenesis [79]. NSCs prepared from this mutant embryonic brain have poorly formed neurospheres. Therefore, Tip60 and KAT8 are indispensable for NSC maintenance during development. They might collaborate with each other to maintain a balance between NSC proliferation and differentiation. In addition, massive apoptotic cell death, which may contribute to cerebral hypoplasia, was observed in *KAT8*-deficient brains but not in *Tip60*-deficient brains. The dysregulation of KAT8 targets might be highly sensitive to the initiation of a program for apoptotic cell death. The deregulation of the epigenetic control in neural cells caused by the loss of KAT8 causes widespread metabolic defects, which leads to abnormal pericyte activation and a breakdown of the vasculature in *KAT8*-deficient brains [80]. This result might reflect phenotypic differences of brains between Tip60 and KAT8 deficiency. Recently, it has been reported that Tip60-mediated H2A.Z acetylation is important for neuronal fate specification using an in vitro culture system [81]. Relatively small sets of genes in which promoters are regulated by bivalent modification could not be activated in *Tip60*-deficient mice during neurogenesis.

It has been reported that other MYST family HATs are also crucial factors for neurogenesis, and their mutation causes neurodevelopmental disorders. KAT6B/MORF was first identified as a gene that is important in neural development by the gene trap method [82]. KAT6B is highly expressed in the developing and adult brain [82,83,84]. KAT6B expression is highest in the SVZ in which NSCs reside, and NSCs derived from *KAT6B*-mutant brains generated fewer colonies of neurospheres [85]. Similar to Tip60 and KAT8, this indicates that KAT6B has a pivotal role in NSC maintenance and neurogenesis. Heterozygous mutations of *KAT6B* in humans cause Genitopatellar syndrome (GPS), a developmental disorder with intellectual disability [86,87]. Homozygous *KAT6A/MOZ* knockout mice show embryonic lethality accompanying a severe deficit of hematopoietic stem cells (HSCs) [88,89]. Interestingly, KAT6A is required for silencing p16^INK4a^ expression in NSCs in order to protect stem cells from prematurely entering replicative senescence [90]. Because Wichmann et al. suggest that cell growth arrest by Tip60 deficiency in MEFs is independent of p16^INK4a^ [59], the underlaying molecular mechanisms in NSC maintenance might be different between Tip60 and KAT6A. Mutations in the *KAT6A* gene relating to developmental disorders with intellectual disability are also reported [91]. Bromodomain- and PHD finger-containing protein 1 (BRPF1) is an activator of KAT6A, KAT6B, and KAT7/HBO1 [92]. Cerebrum-specific *BRPF1*-deficient mice using the *Emx-Cre* line show hypoplasia of the dentate gyrus and dysregulation of neuronal migration, cell cycle progression, and transcription of key genes, which are important for hippocampus development [93,94]. The phenotypes of cerebrum-specific *BRPF1*-deficient mice look less severe than those of Tip60 deficiency, suggesting MYST family HATs might have specific functions in neurodevelopment and neurogenesis. The mutations of the *BRPF1* gene in humans are also related to neurodevelopmental disorders with syndromic intellectual disability [95]. These mutations are associated with impairment of histone H3K23 propionylation [7]. Because global inactivation of *KAT7/HBO1* is embryonic lethal [96], the precise role of KAT7 in brain development and neurogenesis remains unknown so far. KAT7 mRNA is highly expressed in the adult brains of mice [96], suggesting a specific role for KAT7 in the brain. It has been reported that KAT7 is required for HSC quiescence and is important for HSC maintenance and self-renewal in adult hematopoiesis [97]. KAT7 might also be involved in NSC maintenance and neurogenesis. These results show that MYST family HATs are crucial for brain development, neurogenesis, and brain function. This warrants further investigation about the role of this family in the brain.

Collectively, we have found the critical role of Tip60 in NSC maintenance and neurogenesis in mice. We also provide evidence that Tip60 is important for switching from neurogenesis to gliogenesis and proper neuronal migration. This observation provides a comprehensive understanding of the importance of epigenetic regulation for brain development and the complex pathophysiology of intellectual disability in humans.

## 4. Materials and Methods

### 4.1. Animals

The mouse *Tip60* genomic fragment was kindly provided by Dr. John Lough at Wisconsin University. Approximately 1.3 and 1.7 kb of the genomic region of *Tip60* were inserted into pBluescript SK containing *Pgk1-Neo* for positive selection and diphtheria toxin A for negative selection (KO II vector) [98,99]. A *loxP* was inserted between exons 9 and 10, and an *Frt–Pgk1-Neo–Frt–loxP* cassette was inserted between exons 11 and 12 to frank exons containing a part of the MYST domain, including the acetyl-CoA binding site with *loxP* sequences (Figure S1A). The linearized targeting construct was electroporated into E14tg2a embryonic stem (ES) cells, which are derived from 129/Ola strain mice. ES cell clones were selected in ES cell culture media supplemented with 200 µg/mL of G418. Targeted clones were screened by Southern blot analysis using 5′ and 3′ probes (Figure S1). Three of the correctly targeted clones were expanded and injected into C57BL/6J blastocysts. Chimeric males were bred to C57BL/6J females to obtain heterozygous *Tip60*-targeted mice. In removing the *Pgk1-Neo* cassette, pCAGGS-FLPe was injected into one-cell stage embryos derived from C57BL/6J females mated with heterozygous *Tip60* targeted males, and the embryos were transplanted into the oviducts in ICR mice. The removal of the *Pgk1-Neo* cassette was confirmed by PCR using mouse tail DNA. This mouse line, which contains a floxed *Tip60* allele without a *Pgk1-Neo* cassette (F allele), was used in this study. The *Tip60^F/+^* mouse was backcrossed to the C57BL/6 mouse at least 6 times.

In order to delete *Tip60* from NSCs, *Nestin-Cre^+^* (*Nes-Cre^+^*) mice, in which Cre recombinase is expressed in NSCs after embryonic day 11.5 (E11.5), were crossed with *Tip60^F/+^* mice. Homozygous floxed *Tip60* females (*Tip60^F/F^*) were crossed with *Tip60^F/+^;Nes-Cre^+^* males to produce mice, in which the *Tip60* genes were knocked out in the NSCs (*Tip60^F/F^;Nes-Cre^+^* indicated as *Tip60* cKO hereafter). All genotypes were confirmed by PCR using primers listed in Supplementary Table S1. All experiments were performed using *Tip60* cKO mice with their littermates as controls. The mice were maintained under controlled environmental conditions consisting of a 12 h/12 h light and dark cycle with free access to standard mouse chow and water.

### 4.2. Histology and IHC

Histological analyses were performed as previously described [100,101]. Newborn pups were fixed in 4% paraformaldehyde/PBS (PFA) at 4 °C overnight, dehydrated, and embedded in paraffin wax. Serial sections (4 µm) were prepared and stained with hematoxylin–eosin. Images were captured using a KEYENCE BZ-X700 (Keyence, Osaka, Japan).

For IHC, each embryo was isolated and perfused with 4% PFA in PBS injected into the left cardiac ventricle. The brain was removed and stored in 4% PFA overnight and equilibrated with cryoprotection solution (20% sucrose in PBS). Brains were embedded in OCT compound, and the coronal sections were prepared using a cryostat (Leica CM-3050S) set at a 14-mm thickness and mounted onto adhesive glass slides (Matsunami MAS-01, Osaka, Japan). For antigen retrieval, sections were soaked in 10 mM of citrate (pH6.0), boiled at 105 °C for 5 min in an autoclave, and kept at room temperature for 30 min. Then, the slides were permeabilized with 0.2% Triton X-100 in PBS and blocked with 5% FBS/0.1% Triton X-100 in PBS (blocking buffer). The sections were incubated with antibodies in blocking buffer at 4 °C overnight. Secondary antibodies were incubated with the sections in blocking buffer at room temperature for 1 h. DAPI (1 μg/mL in PBS) was used for nuclear staining. The sections were mounted with ProLong Gold antifade reagent (ThermoFisher, Waltham, MA, USA), and images were captured using a KEYENCE BZ-X700. Image analysis was performed with a Cell Analyzer (Keyence). The antibodies used were as follows: MAP2 (1:250, Proteintech, Rosemont, IL, USA, 17490-1-AP), GFAP (1:2000 Sigma, St. Louis, MI, USA, G3893), Tbr1 (1:500 Abcam, Cambridge, UK, ab31940), Ctip2 (1:1000 Abcam ab18465), Cux1 (1:500 Santa Cruz sc-13024), Tbr2 (1:1000 Abcam ab183991), Pax6 (1:1000 MBL PD022), histone H3S10P (1:1000 Millipore, Burlington, MA, USA, 05-806), activated caspase 3 (1:2000 CST 9665), Iba1 (1:1000 Abcam ab178846), GLP-1 (1:250 Santa Cruz sc-57166), H4K8Ac (1:1000 MBL MABI0408), H4K12Ac (1:500 Abcam ab1761), γH2AX (1:500 Upstate 05-636), γH2AX (1:500 Cell Signaling, Danvers, MA, USA, 2577), p21 (1:500 BD Sciences, Franklin Lakes, NJ, USA, 556431), and 53BP1 (1:1000 BETHYL A300-273A).

For the detection of growing cells in embryos, pregnant mice were intraperitoneally injected with BrdU (10 mg/mL in PBS) at 100 µg/g body weight, and mice were sacrificed after 2 h [102]. Embryos were isolated from the uterus, and perfusion was performed with 4% PFA in PBS injected into the left cardiac ventricle. Dissected brains were equilibrated with cryoprotection solution (20% sucrose in PBS) and embedded in the OCT compound. Sections (14-μm thickness) were antigen retrieved with 10 mM of citrate (pH6.0) and boiled at 105 °C for 5 min in an autoclave. Incorporated BrdU in embryos was detected by incubating with an anti-BrdU antibody (1:200 BD Sciences 347580) overnight at 4 °C, following treatment with a secondary antibody (AlexaFluor488 1:1000 ThermoFisher) at room temperature for 1 h, and the sections were counterstained with DAPI. Images were captured using a KEYENCE BZ-X700. Ten different areas per embryo were analyzed. Statistical analysis was determined by an unpaired *t*-test.

### 4.3. NSC Culture

The cerebral cortices of mouse embryos at gestational day 14.5 (E14.5) were dissected and mechanically triturated with a P1000 micropipette into single cells as previously described [100,101,103]. The cells were seeded into 60-mm low-attachment PrimeSurface dishes (Sumilon, Sumitomo Bakelite, Tokyo, Japan) at a density of 1 × 10^6^ cells/dish and cultured in neural stem cell-growing medium containing NeuroBasal medium, 2% B-27 without vitamin A, 2 mM glutamine, 20 ng/mL of rhEGF (Pepro Tech, Cranbury, NJ, USA), and 10 ng/mL of rhFGF2 (Pepro Tech) in a humidified incubator containing 5% CO_2_. After 4–5 days, the formed neurospheres were collected and used for further experiments.

### 4.4. BrdU Incorporation Assay

Neurospheres were treated with Accumax (Innovative Cell Technologies, Inc., San Diego, CA, USA) at room temperature for 10 min and dispersed into single cells using a P200 micropipette. The dispersed cells (2 × 10^5^ cells/well) were seeded into 12-well plates (Iwaki, Tokyo, Japan) with ploy-L-ornithine coated coverslips and cultured in neural stem cell growth medium overnight. The cells were incubated with 10 μM BrdU in the medium for 2 h and then fixed with 70% ethanol for 20 min. The cells were treated with 2.5 N HCl for 20 min, the coverslips were blocked in PBS containing blocking buffer and incubated with anti-BrdU antibody (1:200 BD Sciences 347580) overnight at 4 °C. The next day, the coverslips were treated with secondary antibody (AlexaFluor488 1:1000 ThermoFisher) at room temperature for 1 h, and then DAPI was used for nuclear staining. Coverslips were mounted with ProLong Gold antifade reagent (ThermoFisher), and the images were captured with a KEYENCE BZ-X700 microscope. Image analysis was performed using Cell Analyzer software (Keyence).

### 4.5. Differentiation Assay

Neurospheres formed from a primary culture were treated with Accumax (Innovative Cell Technologies, Inc.) at room temperature for 10 min and dispersed into single cells using a P200 micropipette. The dispersed cells (2 × 10^5^ cells/well) were seeded into 12-well plates (Iwaki) containing ploy-L-ornithine coated coverslips and cultured in a differentiation medium containing NeuroBasal medium, 2% B-27 with Vitamin A, 2 mM glutamine, and 1% FBS, and incubated for 7 days. The cells were fixed with 4% paraformaldehyde (PFA) in PBS for 10 min and blocked for 1 h in a blocking buffer. The coverslips were incubated with primary antibodies (Tuj1/beta-III Tubulin 1:2000 Abcam ab1820, GFAP 1:1000 Sigma G3893) overnight at 4 °C. The next day, the coverslips were treated with secondary antibodies (AlexaFluor488 1:1000 ThermoFisher, Cy3 1:1000 Amersham Pharmacia, Little Chalfont, UK) at room temperature for 1 h, and then DAPI was used for nuclear staining. The coverslips were mounted with a ProLong Gold antifade reagent (ThermoFisher), and images were captured with a KEYENCE BZ-X700 microscope. Image analysis was performed with Cell Analyzer software (Keyence).

### 4.6. Quantitative RT-PCR (qRT-PCR)

Total RNA was isolated from mouse embryonic brains or neurospheres lysed with Trizol reagent (Invitrogen, Waltham, MA, USA). Isolated RNA was treated with RNase-free DNase I (TaKaRa, Tokyo, Japan) to eliminate contaminated DNA, and cDNA synthesis using 1 mg of total RNA was performed with the PrimeScript RT reagent kit (Perfect Real Time, TaKaRa) according to the manufacturer’s instructions.

Real-time PCR was carried out using THUNDERBIRD Next SYBR^®^ qPCR Mix (TOYOBO, Osaka, Japan). Fluorescence was monitored by the Thermal Cycler Dice Real Time System II. Expression levels of target genes were normalized to those of β-actin and relative expression was calculated using the ΔΔCt method. The primers used in this study are listed in Supplementary Table S2.

### 4.7. Western Blots

The cells were washed with PBS and lysed with lysis buffer (20 mM Tris-HCl [pH 7.5], 1% NP-40, 150 mM NaCl, 10% glycerol, and protease inhibitor cocktail set I [Calbiochem]). The lysates were kept on ice for 30 min and centrifuged at 20,000× *g* for 15 min to remove unsolved materials. Protein concentration in the supernatants was determined by the Bradford protein assay (Bio-Rad, Hercules, CA, USA) using BSA as a standard. The total proteins were separated on 10% SDS-PAGE with a running buffer (25 mM Tris, 192 mM glycine, and 0.1% SDS) at 100 V for 90 min. After electrophoresis, the separated proteins were transferred to a nitrocellulose membrane using a wet transfer apparatus with transfer buffer (25 mM Tris, 192 mM glycine, and 20% methanol) at 0.4 A for 60 min. Membranes were blocked in 2% FBS/0.5% skimmed milk in PBST (0.05% Tween 20) for 1 h and then probed with the primary antibody overnight at 4°C. Primary antibodies used were as follows: rabbit anti-Tuj1 (1:2000 Abcam ab1820), mouse anti-GFAP (1:1000 Sigma G38931), and mouse anti-b-actin (1:1000 MBL M177-3). Beta-actin was used as a loading control. Horseradish peroxidase-conjugated secondary antibodies (Cytiva, Marlborough, MA, USA) were used at 1:2000 for 1 h at room temperature. The bands were visualized using an ECL detection system (Cytiva) and analyzed with Amersham ImageQuant 800 (Cytiva).

### 4.8. RNA Sequencing (RNA-Seq) and Data Processing

Brains were isolated from embryos at E14.5, and the meninges were carefully removed. In addition, the dissected cortexes were quickly frozen. Brains from three *Tip60* cKO mice and three control mice were used for RNA-seq analysis. Total RNA was extracted with Trizol reagent (Invitrogen), and sequencing libraries were constructed with the Illumina TruSeq Stranded Total RNA LT w/RiboZero Kit. Moreover, 2 × 75 base paired-end sequencing was performed using an Illumina NextSeq 500 instrument. RNA-seq data were mapped to the GRCm38/mm10 genome reference using the Illumina BaseSpace app TopHat alignment (ver. 1.0.1). Gene-based reads per kilobase of exon per million mapped reads (RPKM) were obtained using AltAnalyze [104] (ver. 2.1.0, http://altanalyze.org/, accessed on 1 October 2020) with default parameters. Genes with protein-coding annotations were considered for further evaluation. PCA was run using the R “prcomp()” function on the 20,079 protein-coding genes. PC1 and PC2 plots were visualized by “ggfortify” in R. Based on volcano plot analysis, genes with insufficient expression (RPKM < 1.0 in either the KO or control groups) were filtered out before the analysis, and we set an adjusted *p* value < 0.05 and fold changes ≥1.5 as the threshold of DE genes in the KO vs. control groups. STRING, a database of protein/gene interactions [105] (https://string-db.org, accessed on 1 December 2022), was used to conduct enrichment analyses on tissue expression data, biological process GO terms, and STRING clusters. Volcano plot, heatmap, and enrichment analyses were visualized using Prism 9 software (ver. 9.5.0, GraphPad, Boston, MA, USA). All the sequencing data used in this work were submitted to the DDBJ DRA (accession number DRA015351).

### 4.9. Statistics

Statistical analyses were performed using a two-tailed *t*-test, and differences were considered significant with *p* values < 0.05.

## Figures and Tables

**Table 1:** The number of pups from *Tip60^F/F^* females mated with *Tip60^F/+^;Nes-Cre+* males. A total of 31 litters were analyzed. The average litter size was 7.4 pups.

	Pups	%	% Expected
*Tip60^F/F^;Nes-Cre+*	56	24.6	25
*Tip60^F/F^;Nes-Cre-*	63	27.6	25
*Tip60^F/+^;Nes-Cre+*	52	22.8	25
*Tip60^F/+^;Nes-Cre-*	57	25.0	25
Total	228	100	100

## Data Availability

DRA015351 for RNA-seq data.

## Supplementary Materials

The supporting information can be downloaded at: https://www.mdpi.com/article/10.3390/ijms24032113/s1.
