# Supplementary material for: Tip60/KAT5 Histone Acetyltransferase Is Required for Maintenance and Neurogenesis of Embryonic Neural Stem Cells
Source: Int J Mol Sci. 2023 Jan 20;24(3):2113. doi: 10.3390/ijms24032113 (PMC9916716; doi:10.3390/ijms24032113)
Supplement: Supplementary file 1 [file ijms-24-02113-s001.zip › ijms-2172670-supplementary.pdf]

Supplementary Figures

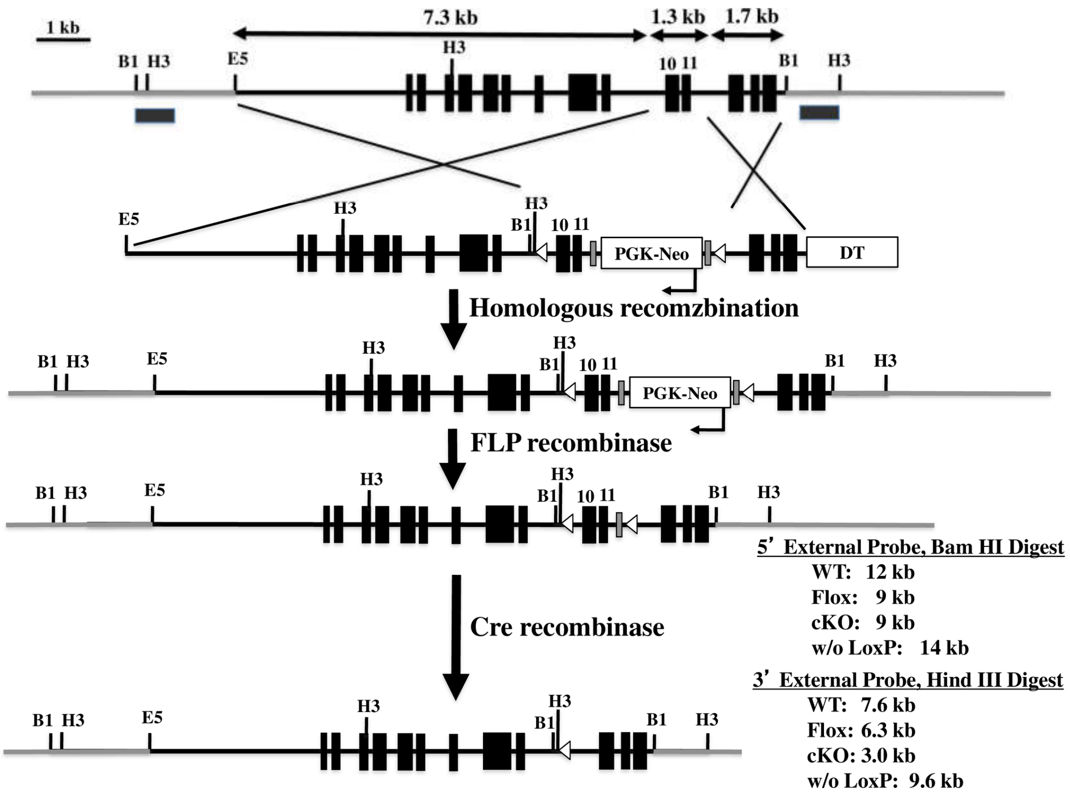

Figure S1. Generation of *Tip60*-targeted allele. Strategy of targeting conditional allele of the *Tip60* gene. A *loxP* sequence and an *Frt–PGK1-Neo–Frt–loxP* cassette were inserted in intron 9 and intron11, respectively. DT, diphtheria toxin fragment A. Targeted ES clones were screened by Southern blot using the 5′ external probe and 3′ external probe. DNAs from neomycin-resistant ES cells were digested with Bam HI for the 5′ probe and Hind III for 3′ probe. A total of 12 kb WT and/or 9 kb targeted bands were detected by the 5′ probe and 7.6 kb WT, and/or 6.3 kb targeted bands were detected by the 3′ probe.

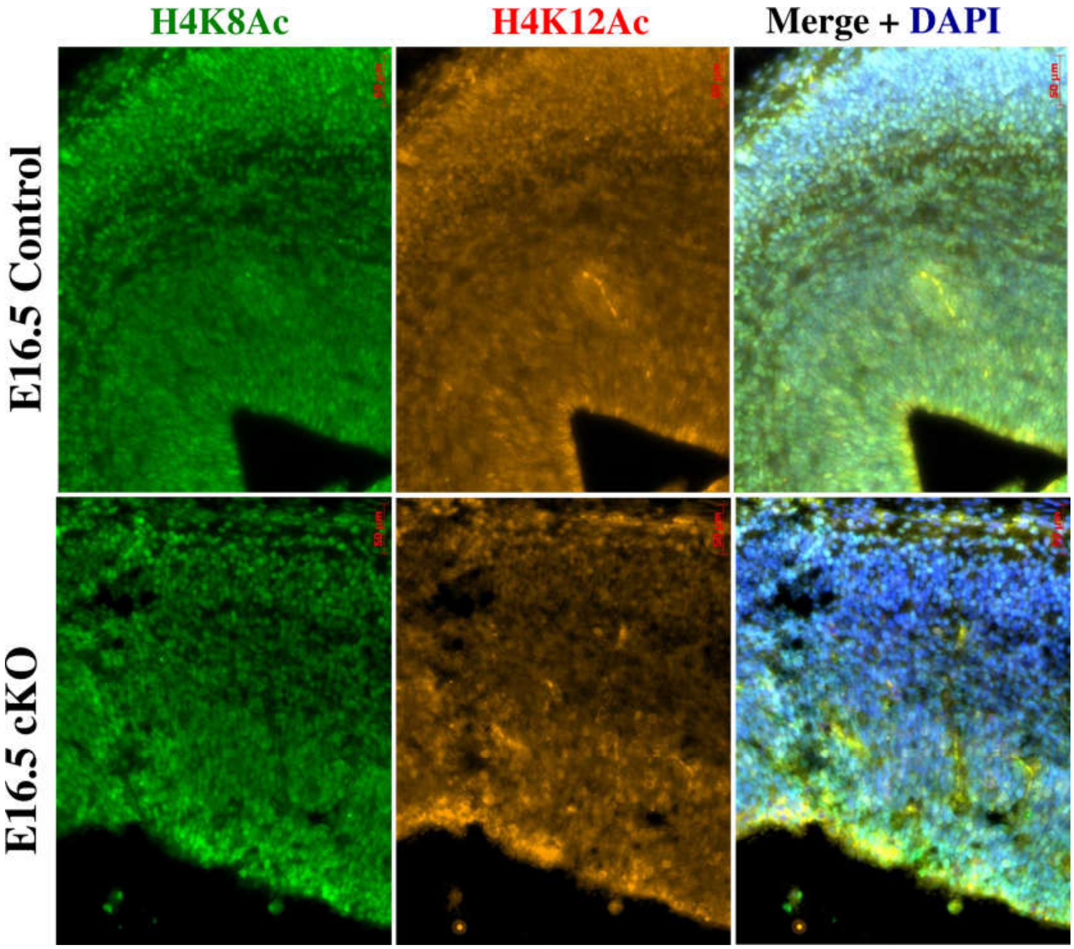

Figure S2. Immunostaining for acetylated histone H4 in E16.5 brains. The brain sections were immunostained for acetylated histone H4 at Lys8 (H4K8Ac) and at Lys12 (H4K12Ac), and DAPI was used for nuclear staining. Scale bars, 50 µm. The staining levels in the cerebral cortex from *Tip60* cKO brain by anti-H4K8Ac and anti-H4K12Ac antibodies is weaker compared with control. This result was confirmed by two independent experiments.

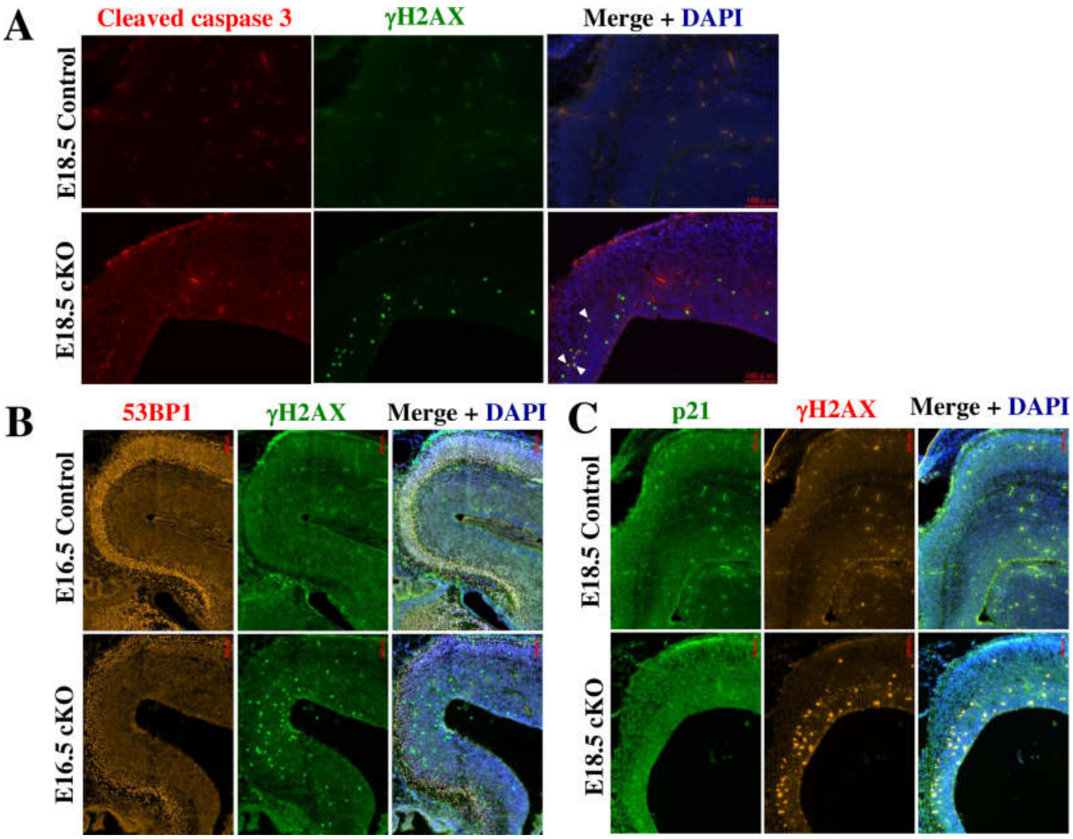

Figure S3. Accumulated DNA damage is easily detected in *Tip60* cKO embryonic brains but not apoptosis and p21. (**A**) Representative images of brains at E18.5 stained for cleaved caspase 3 (apoptosis) and γH2AX (DNA damage) by immunofluorescence. DAPI was used for nuclear staining. Scale bars, 100 μm. Arrow heads indicate co-stained cells. (**B**) Representative images of brains at E16.5 stained for 53BP1 and γH2AX and DNA damage markers by immunofluorescence. DAPI was used for nuclear staining. Scale bars, 100 μm. γH2AX-positive cells in *Tip60* cKO brain are already accumulated at the earlier stage. In addition, 53BP1-positive cells co-localized with γH2AX cannot be detected. (**C**) Representative images of the brain at E18.5 stained for p21 and γH2AX by immunofluorescence. DAPI was used for nuclear staining. Scale bars, 100 μm. Cellular senescence-related p21, which is induced in response to DNA damage, is not accumulated in γH2AX-positive cells in *Tip60* cKO brain. All staining results were confirmed by at least two independent experiments.

Supplementary Tables

Supplementary Table S1. Primer sequences for genotype.

**Target Gene**	**Forward Primer**	**Reverse Primer**
Tip60	CACGGACTCTTAGCTCTGACAC	GCCATATTTGCCTCTAGTTAGGAA
Tip60-KO	AGTACTGTAGGGAAAATGCCTGAG	
Cre	CCGGGCTGCCACGACCAA	GGCGCGGCAACACCATTTTT

Supplementary Table S2. Primer sequences for qRT-PCR analysis.

**Target Gene**	**Forward Primer**	**Reverse Primer**
Tip60	CCAAGGAAAAGGAATCCACA	CAAGATGGTTTGGGACCAGT
CD11b	GGCTCCGGTAGCATCAACAA	ATCTTGGGCTAGGGTTTCTCT
CX3CR1	GAGTATGACGATTCTGCTGAGG	CAGACCGAACGTGAAGACGAG
IBA1	ATCAACAAGCAATTCCTCGATGA	CAGCATTCGCTTCAAGGACATA
TMEM119	CCTACTCTGTGTCACTCCCG	CACGTACTGCCGGAAGAAATC
Gcg	TGCTGAAGGGACCTTTACCAGTGA	GCCTTTCACCAGCCAAGCAATGAA
β-actin	CGTCGACAACGGCTCCGGCATG	GGGCCTCGTCACCCACATAGGAG
